# ﻿A new species of the newt genus *Hypselotriton* (Amphibia, Urodela, Salamandridae) from Jiangxi Province, southeastern China

**DOI:** 10.3897/zookeys.1208.126092

**Published:** 2024-08-05

**Authors:** Zhihao Jiang, Song Huang, Qiangyong Fan, Lin Cheng, Yanan Gong, Zhangbo Cui, Tierui Zhang, Wenjun Lan, Zhian Zou, Xuanzhi Huang, Jean Raffaëlli, Jinmin Chen

**Affiliations:** 1 The Anhui Provincial Key Laboratory of Biodiversity Conservation and Ecological Security in the Yangtze River Basin, College of Life Sciences, Anhui Normal University, Wuhu 241000, China Anhui Normal University Wuhu China; 2 Jiangxi Wuyishan National Nature Reserve Administration Bureau, Wuyishan National Nature Reserve, Shangrao, Jiangxi, China Wuyishan National Nature Reserve Shangrao China; 3 Penclen, Plumelec, 56420, France Unaffiliated Plumelec France

**Keywords:** Black patches, fire-bellied newts, geographical isolation, morphology, phylogenetics, taxonomy

## Abstract

A new newt species, *Hypselotritonhuanggangensis***sp. nov.**, is described based on nine specimens collected from Huanggangshan Mountains, Yanshan County, Jiangxi, China. Morphologically, the new species is characterized by the combination of nine external characters: (1) obvious black patches with clear boundaries on the whole body; (2) ground color of the dorsal body tan; (3) ground color of venter bright orange; (4) skin rough; (5) vertebral ridge weak; (6) fingers and toes overlapping when forelimb and hindlimb adpressed towards each other along body; (7) postocular orange spot absent; (8) small white warty glands around the eye; (9) two discontinuous longitudinal lines formed by white warty glands from neck to lateral parts of tail. Molecularly, the new species forms an independent clade with strong support in the phylogenetic trees of the genus based on the mitochondrial locus of NADH dehydrogenase subunit 2 (ND2) gene fragments. The new species distinctly differs from *H.fudingensis* by differences in its body measurements, vertebral ridge, dorsal black patches, and ventral black patches. Furthermore, the new species and *H.fudingensis* are geographically isolated by a series of high mountain ranges, including the Wuyishan and Jiufengshan Mountains. The number of *Hypselotriton* species is now 11.

## ﻿Introduction

The newt genus *Hypselotriton* Wolterstorff, 1934 (Urodela, Salamandridae) is distributed in China, including Anhui, Jiangsu, Zhejiang, Fujian, Jiangxi, Guangdong, Hubei, Henan, Hunan, Yunnan, and Guizhou Provinces (AmphibiaChina 2024; AmphibiaWeb 2024; Frost 2024). The generic classifications between *Hypselotriton* and *Cynops* has a lengthy history of taxonomic debate (Tschudi and Johann 1838; Wolterstorff 1934; Chang 1935; Zhao and Hu 1984; Zhao et al. 1988; Chan et al. 2001; Weisrock et al. 2006; Zhang et al. 2008; Dubois and Raffaëlli 2011, 2012; Raffaëlli 2013, 2022; Tominaga et al. 2013; Fei and Ye 2016). Previously, some arrangements suggested that *Hypselotriton* is a junior synonym of *Cynops* (Ye et al. 1993; Wu et al. 2010a; Fei and Ye 2016). However, recent phylogenetic studies have presented evidence that *Cynops* sensu lato (including *Hypselotriton*) is paraphyletic with respect to *Pachytriton* and *Paramesotriton* (Rancilhac et al. 2021; Zhong et al. 2021; Yuan et al. 2022). Following the latest taxonomic arrangements (Dubois and Raffaëlli 2009; Dubois et al. 2021; Frost 2024) and the premise of monophyly, the genus *Cynops* Tschudi, 1838 is restricted to the Japanese species and all Chinese species are placed in the genus *Hypselotriton*.

Currently, the following 10 species of *Hypselotriton* have been recorded: *H.cyanurus* Liu, Hu & Yang, 1962; *H.yunnanensis* Yang, 1983; *H.chenggongensis* Kou & Xing, 1983; *H.wolterstorffi* Boulenger, 1905; *H.orientalis* David, 1873; *H.orphicus* Risch, 1983; *H.fudingensis* Wu, Wang, Jiang & Hanken, 2010; *H.maguae* Lyu, Qi & Wang, 2023; *H.jiaoren* Lyu, Qi & Wang, 2023; *H.glaucus* Yuan, Jiang, Ding, Zhang & Che, 2013. Recent studies suggest that overall species richness of *Hypselotriton* is underestimated (Yuan et al. 2022; Lyu et al. 2023). The reevaluations of the “widespread” species (*H.orientalis* and *H.yunnanensis*) and the survey of unexplored areas are likely to reveal overlooked diversity.

Huanggangshan Mountains (about 10 km long) is located on the northwestern side of the Wuyishan Mountains (about 550 km long), China. The highest peak (2161 m a.s.l) of Huanggangshan Mountains is known as the “roof of Eastern Mainland China” and “the first peak in the southeast of the mainland” (Lin and Ye 1985). During the recent surveys, on the northwestern side of the Huanggangshan Mountains, nine fire-bellied newts of unidentified *Hypselotriton* population were sampled in a small waterhole. After examination, they were found to differ from other congeneric members in both morphological and molecular characteristics. As a result, we herein describe it as a new species of *Hypselotriton*.

## ﻿Materials and methods

### ﻿Sampling

**Figure 1.**
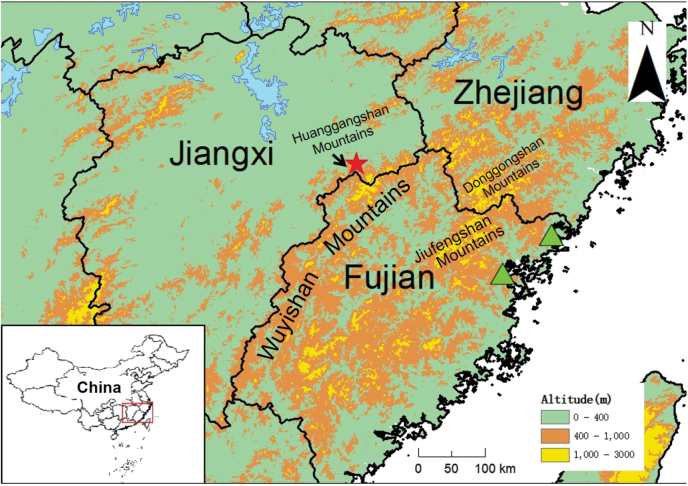
Geographic distribution of *Hypselotritonhuanggangensis* sp. nov. (red star) and *H.fudingensis* (green triangles) in southeastern China. They are separated by the Wuyishan and Jiufengshan Mountains.

Nine specimens were collected in a small waterhole (28.15°N, 117.53°E; elevation 84 m) from Huanggangshan Mountains, Yanshan County, Shangrao City, Jiangxi (Fig. 1). The tiptoes of the specimens (the first toe of each specimen) were cut off and immediately preserved in 75% ethanol. These samples were then used for DNA analysis. After identifying that it is a new species, all fire-bellied newts were humanly euthanized by the injection of 0.7% tricaine methanesulfonate (MS222) solution (Yang et al. 2023), and fresh liver tissue was extracted and immediately preserved in 95% ethanol. The specimens were fixed in 10% formalin for one day, subsequently preserved in 75% ethanol and deposited in Anhui Normal University Museum (voucher numbers: HSA23097–23103, HSA23075–23076). Collections of all animals used for this present study obey the Wildlife Protection Act of China, following the guidelines and regulations approved by the internal review board of AHNU (approval no. AHNU-ET2023110), and with the permissions of local government authorities.

### ﻿Morphological examination

External measurements were made for the seven specimens of *H.huanggangensis* sp. nov. and nine specimens of *H.fudingensis* with digital calipers to the nearest 0.1 mm. Only adult specimens were measured (Lyu et al. 2023). These 14 measurements are as follows: total length (TOL) from tip of snout to tip of tail; snout–vent length (SVL) from tip of snout to posterior edge of vent; tail length (TAL) from posterior edge of vent to tip of tail; maximum tail depth (TAD); head length (HL) from tip of snout to the posterior edge of the parotoid gland; maximum head width (HW); snout length (SL) from tip of snout to the anterior corner of eye; eye diameter (ED) from the anterior corner to the posterior corner of the eye; interorbital distance (IOD) between the anterior corner of each eye; eye–nostril length (EN) from the anterior corner of the eye to the nostril; internasal distance (IND) between the external nares; axilla–groin length (AG) between the axilla and the groin along the body; forelimb length (FLL) from elbow to tip of finger III; and hindlimb length (HLL) from knee to tip of toe III.

Statistical analyses on the morphometric measurements were performed in IBM SPSS Statistics 27.0. Males and females were analyzed separately, due to obvious sexual size dimorphism (Fei et al. 1990, 2006, 2012; Fei and Ye 2016). All measurements were made to normalize and reduce the variance (most *P* values > 0.05 in the Levene’s test). Univariate Analysis of Covariance (ANCOVA) with SVL as the covariate were used to test for differences between *H.huanggangensis* sp. nov. and *H.fudingensis* (Lai & Lue, 2008).

### ﻿Molecular phylogeny

Total genomic DNA was extracted from ethanol-preserved liver tissues, using the Qiagen DNEasy blood and tissue extraction kit (Qiagen Inc., Valencia, CA, USA). The phylogenetic relationships within *Hypselotriton* were derived from an analysis of the mtDNA fragment that codes for subunit two of NADH dehydrogenase (ND2) and its flanking tRNAs. A 1026-bp fragment was amplified using primers KIZL4437 (Yuan et al. 2011) and 5081R (Wu et al. 2010b). Two internal primers ND2–38R (5′–TATTCAYCCTAARTGTGCR–3′) and 4416F (Wu et al. 2010b) were applied for sequencing (Yuan et al. 2013). Standard polymerase chain reactions (PCR) were performed in a final volume of 15 ul with the following procedures: initial denaturation at 94 °C for 5 min, 35 amplification cycles at 94 °C for 1 min, annealing for 1 min at 52 °C, extension for 1 min at 72 °C. Final extension at 72 °C was conducted for 10 min. The successfully amplified products were purified using ExoSAP-IT purification kit according to the manufacturer’s instruction (Yuan et al. 2013). Purified PCR products were directly sequenced in both directions using a BigDye Terminator Cycle Sequencing Kit (v. 2.0, Applied Biosystems, Foster City, California, USA) and an ABI PRISM 3730 automated DNA sequencer (Yuan et al. 2013). For the phylogenetic analyses, 38 sequences from additional Chinese *Hypselotriton* congeners and two sequences of outgroup species of the genera *Pachytriton* Boulenger, 1878 and *Paramesotriton* Chang, 1935, were obtained from GenBank and incorporated into our dataset. Detailed information is provided in Table 1 (ID means the ordinal numbers of the species). DNA sequences were aligned using MEGA v. 6.0.6 (Kumar et al. 2018) with default parameters and manually checked.

**Table 1.** Localities, voucher information (Holotype: HSA23097), and GenBank accession numbers for all samples of ND2 used in this study.

ID	Species	Localities	Voucher	ND2
1	*Hypselotritonhuanggangensis* sp. nov.	China: Jiangxi: Shangrao: Yanshan	HSA23075	PP590780
2	*Hypselotritonhuanggangensis* sp. nov.	China: Jiangxi: Shangrao: Yanshan	HSA23076	PP590788
3	*Hypselotritonhuanggangensis* sp. nov.	China: Jiangxi: Shangrao: Yanshan	HSA23097	PP590781
4	*Hypselotritonhuanggangensis* sp. nov.	China: Jiangxi: Shangrao: Yanshan	HSA23098	PP590782
5	*Hypselotritonhuanggangensis* sp. nov.	China: Jiangxi: Shangrao: Yanshan	HSA23099	PP590783
6	*Hypselotritonhuanggangensis* sp. nov.	China: Jiangxi: Shangrao: Yanshan	HSA23100	PP590784
7	*Hypselotritonhuanggangensis* sp. nov.	China: Jiangxi: Shangrao: Yanshan	HSA23101	PP590785
8	*Hypselotritonhuanggangensis* sp. nov.	China: Jiangxi: Shangrao: Yanshan	HSA23102	PP590786
9	*Hypselotritonhuanggangensis* sp. nov.	China: Jiangxi: Shangrao: Yanshan	HSA23103	PP590787
10	*Hypselotritonorientalis*	China: Anhui: Huangshan: Furonggu	SYS a002711	OQ116690
11	*Hypselotritonorientalis*	China: Anhui: Huoshan: Shangtushi	KIZ 021844	ON793742
12	*Hypselotritonorientalis*	China: Anhui: Xiuning: Dafu	KIZ 021962	ON793737
13	*Hypselotritonorientalis*	China: Henan: Xinyang: Mt Jigong	KIZ 013021	ON793736
14	*Hypselotritonorientalis*	China: Jiangxi: Jiujiang	KIZ 020539	ON793739
15	*Hypselotritonorientalis*	China: Jiangxi: Shangrao: Wannian	CIB 97867	GU301788
16	*Hypselotritonorientalis*	China: Jiangxi: Shangrao: Wuyuan	KIZ YPX25002	ON793740
17	*Hypselotritonorientalis*	China: Zhejiang: Jinhua	KIZ 06358	ON793718
18	*Hypselotritonorientalis*	China: Zhejiang: Quzhou	CIB 97919	GU301790
19	*Hypselotritonorientalis*	China: Zhejiang: Taizhou: Tiantai	KIZ 012941	ON793732
20	*Hypselotritonfudingensis*	China: Fujian: Ningde: Mt Taimu	CIB 97874	GU301785
21	*Hypselotritonfudingensis*	China: Fujian: Ningde: Jiulongjing	SYS a008487	OQ116688
22	*Hypselotritonfudingensis*	China: Fujian: Ningde: Jiulongjing	SYS a008488	OQ116689
23	*Hypselotritonfudingensis*	China: Fujian: Ningde: Qingyu	KIZ 012214	ON793743
24	*Hypselotritonglaucus*	China: Guangdong: Meizhou: Mianyang	KIZ 09793	ON793715
25	*Hypselotritonglaucus*	China: Guangdong: Meizhou: Mianyang	KIZ 09799	ON793716
26	*Hypselotritonglaucus*	China: Guangdong: Meizhou: Mianyang	KIZ 09800	ON793717
27	*Hypselotritonorphicus*	China: Fujian: Fuzhou: Yongtai	KIZ 09905	ON793728
28	*Hypselotritonorphicus*	China: Fujian: Quanzhou: Mt Daiyun	KIZ 09839	ON793723
29	*Hypselotritonorphicus*	China: Guangdong: Chaozou	KIZ 09816	ON793719
30	*Hypselotritonjiaoren*	China: Guangdong: Qingyuan: Yingde	SYS a008786	OQ116679
31	*Hypselotritonjiaoren*	China: Guangdong: Qingyuan: Yingde	SYS a008787	OQ116680
32	*Hypselotritonjiaoren*	China: Guangdong: Qingyuan: Yingde	SYS a008788	OQ116681
33	*Hypselotritonjiaoren*	China: Guangdong: Qingyuan: Yingde	SYS a008789	OQ116682
34	*Hypselotritonjiaoren*	China: Guangdong: Qingyuan: Yingde	CIB 118534	OQ116683
35	*Hypselotritonjiaoren*	China: Guangdong: Qingyuan: Yingde	SYS a008791	OQ116684
36	*Hypselotritonmaguae*	China: Jiangxi: Fuzhou: Mt Magu	CIB 118535	OQ116685
37	*Hypselotritonmaguae*	China: Jiangxi: Fuzhou: Mt Magu	SYS a007032	OQ116686
38	*Hypselotritoncyanurus*	China: Guizhou: Liupanshui: Shuicheng	CIB 95897	GU301784
39	*Hypselotritoncyanurus*	China: Guizhou: Liupanshui: Shuicheng	KIZ 02331	ON793754
40	*Hypselotritoncyanurus*	China: Guizhou: Liupanshui: Shuicheng	KIZ 02332	ON793755
41	*Hypselotritonyunnanensis*	China: Yunnan: Chuxiong: Zijing	KIZ 021922	ON793749
42	*Hypselotritonyunnanensis*	China: Yunnan: Chuxiong: Zijing	KIZ 021923	ON793750
43	*Hypselotritonyunnanensis*	China: Yunnan: Kunming: Gulu	KIZ 022160	ON793752
44	*Hypselotritonyunnanensis*	China: Yunnan: Kunming: Huahongdong	KIZ 022157	ON793751
45	*Hypselotritonyunnanensis*	China: Yunnan: Pu’er: Ning’er	KIZ 01445	ON793756
46	*Hypselotritonyunnanensis*	China: Yunnan: Pu’er: Ning’er	KIZ 03900	ON793747
47	*Hypselotritonyunnanensis*	China: Yunnan: Pu’er: Ning’er	KIZ 03901	ON793748
48	*Pachytritonarchospotus*	China: Hunan: Guidong	KIZ 04563	KU375007
49	*Paramesotritonchinensis*	China: Zhejiang: Jinhua:Panan	KIZ 06335	KU375034

The matrilineal genealogy was reconstructed using Bayesian-inference (BI) and maximum-likelihood (ML) methods based on ND2 gene. PartitionFinder2 was used to test the best partitioning scheme and jModelTest v. 2.1.2 was used to test the best fitting nucleotide substitution model. The data were analyzed using BI in MrBayes v. 3.2.4 (Ronquist et al. 2012), and ML in RaxmlGUI v. 1.3 (Silvestro and Michalak 2012). Two independent runs were conducted in a BI analysis, each of which was performed for 10 million generations and sampled every 1000 generations with the first 25% samples discarded as burn-in, resulting in a potential scale reduction factor (PSRF) of <0.005. The analyses used the proportion of invariable sites estimated from the data and 1000 bootstrap pseudoreplicates under the GTR+gamma model (Chen et al. 2021). Nodes in the trees were considered well supported when Bayesian posterior probabilities (BPP) were ≥ 0.95 and ML bootstrap support (BS) was ≥ 70% (Chen et al. 2021). Mean genetic distances between and within species were calculated in MEGA v. 6.0.6 using the uncorrected genetic distance (*p*-distances) model.

## ﻿Results

Morphologically, our newly collected specimens can be distinguished from all known congeners (details in the taxonomic account below), which can be reliably identified by the obvious black patches with clear boundaries on the whole body and weak vertebral ridge. Statistical analyses on the morphometric measurements were performed on the specimens from northeastern Jiangxi and its sister species *H.fudingensis* from northeastern Fujian (Table 2). The results of *T*-test on morphometrics showed that individuals of the northeastern Jiangxi population and *H.fudingensis* are obviously different in HL, ED, and IND for males (*p*-values < 0.05), and in TAD for females (*p*-values < 0.05). Furthermore, once differences attributable to SVL were accounted for (Table 4), there were significant differences between *H.huanggangensis* sp. nov. and *H.fudingensis* for TAD, HL, ED, and AG in males, and significant differences for TOL, TAD, HL, HW, AG, FLL and HLL in females.

**Table 2.** Morphometric comparisons based on the morphometric measurements (in mm) of *Hypselotritonhuanggangensis* sp. nov. and *H.fudingensis*. * *p*-values < 0.05, ** *p*-values < 0.01.

	Holotype	*H.huanggangensis* sp. nov.		*H.fudingensis*		*P*-values	
	HSR23097	Male (*n* = 5)	Female (*n* = 2)	Male (*n* = 2)	Female (*n* = 7)	Males	Females
TOL	75.14	73.0–79.1 (74.8±2.5)	79.6–89.4	69.4–77.7	81.1–101.0 (90.8±6.6)	0.691	0.277
SVL	45.62	42.9–47.3 (44.9±1.7)	44.4–51.1	42.9–45.8	46.5–54.5 (51.0±3.3)	0.754	0.314
TAL	30.35	29.0–33.0 (30.8±1.5)	36.1–38.7	28.8–31.6	36.1–49.0 (40.7±4.4)	0.669	0.263
TAD	7.08	6.7–7.4 (7.0±0.3)	7.4–8.4	5.1–6.2	5.5–7.4 (6.4±0.6)	0.224	0.045*
HL	13.6	12.6–13.6 (13.0±0.4)	12.0–14.4	11.7–12.5	13.2–15.5 (14.1±0.7)	0.040*	0.298
HW	9.16	8.7–9.5 (9.1±0.3)	9.0–10.4	8.3–8.8	8.9–10.5 (9.8±0.5)	0.084	0.878
SL	3.98	4.0–4.4 (4.2±0.2)	3.5–4.6	4.2–4.4	4.1–4.9 (4.4±0.3)	0.472	0.645
ED	3.63	3.5–3.7 (3.6±0.1)	3.7–4.3	3.2–3.3	3.1–4.0 (3.5±0.3)	0.003**	0.202
IOD	5.37	5.0–5.4 (5.3±0.2)	4.9–5.2	4.4–5.3	4.8–5.8 (5.4±0.3)	0.507	0.333
EN	2.86	2.9–3.2 (3.0±0.1)	2.6–3.5	2.9–3.1	2.6–3.3 (3.1±0.2)	0.809	0.914
IND	2.77	2.4–2.8 (2.5±0.2)	2.6–2.9	1.9–2.1	1.9–3.8 (2.5±0.6)	0.021*	0.668
AG	20.19	18.4–21.4 (19.8±1.1)	21.5–24.5	18.2–19.7	20.8–26.1 (24.2±1.9)	0.386	0.659
FLL	12.28	12.3–14.5 (13.4±1.0)	13.6–15.2	12.9–14.2	13.3–15.2 (14.2±0.8)	0.807	0.780
HLL	13.95	14.0–16.2 (15.0±1.0)	13.8–17.2	14.0–14.4	13.5–16.3 (14.9±1.1)	0.188	0.878

**Figure 2.**
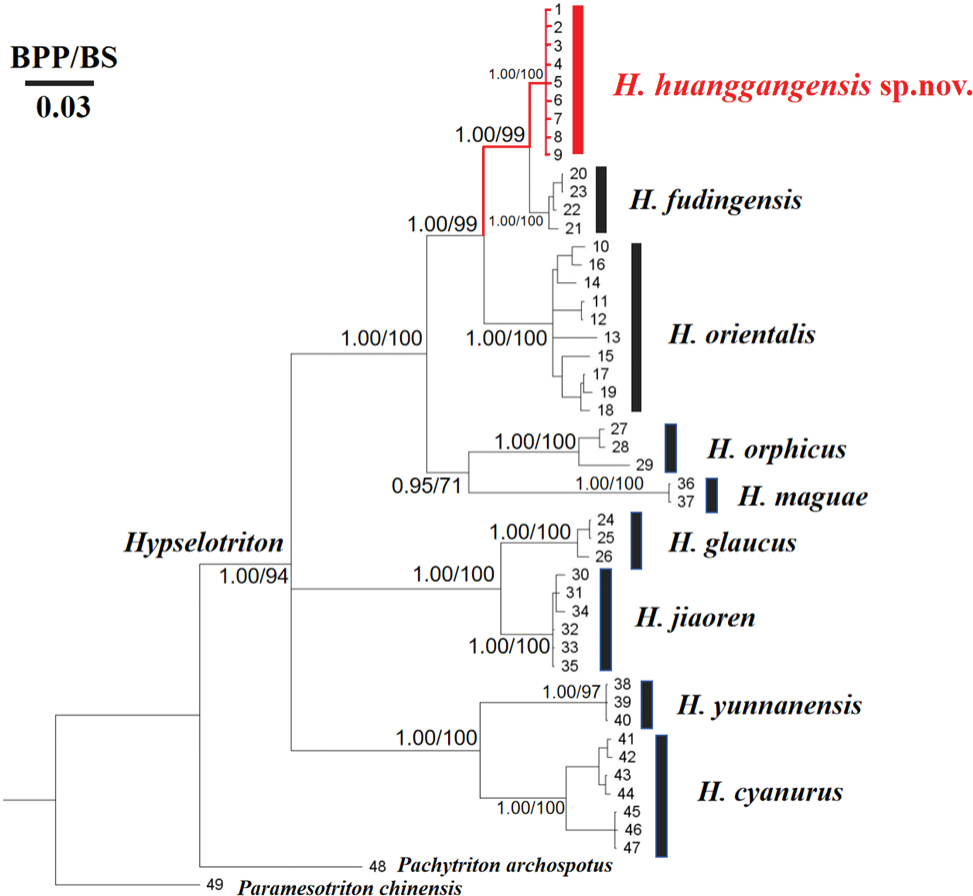
Bayesian-inference tree and maximum-likelihood phylogenies based on mitochondrial ND2 gene. Bayesian posterior probabilities and the bootstrap supports (BPP/BS) are shown near the notes. Number at the ends of the lineages correspond to the IDs in Table 1.

BI and ML analyses resulted in similar identical topologies (Fig. 2). As shown in the tree (Fig. 2), three major clades with strong support were revealed for the samples of *Hypselotriton*, while the relationship among these clades are not resolved. The first clade is composed of samples of *H.cyanurus* (BPP 1.00, BS 100) and *H.yunnanensis* (BPP 1.00, BS 97). The second clade consists of *H.glaucus* from eastern Guangdong (BPP 1.00, BS 100) and *H.jiaoren* from northern Guangdong (BPP 1.00, BS 100). In the third clade, the new specimens from northeastern Jiangxi form a distinct lineage (BPP 1.00, BS 100), which is sister to *H.fudingensis* with support values (BPP 1.00, BS 99). The genetic distances based on the ND2 gene among species of *Hypselotriton* are presented in Table 3. The putative new species from Huanggangshan Mountains showed obvious genetic divergence from other congeners. When compared with closely related recognized congeners, the minimum uncorrected genetic distance was 2.2% between the clade from Huanggangshan Mountains and *H.fudingensis* (Table 3).

**Table 3.** Uncorrected *p*-distances (%) based on the ND2 gene among *Hypselotriton* species (in 0.1%).

ID	Species	1	2	3	4	5	6	7	8	9
1	*Hypselotritonhuanggangensis* sp. nov.	0								
2	*Hypselotritonorientalis*	7.2	2.7							
3	*Hypselotritonfudingensis*	2.2	8.0	0.5						
4	*Hypselotritonglaucus*	18.8	19.6	18.7	0.5					
5	*Hypselotritonorphicus*	11.8	14.0	12.3	19.9	2.0				
6	*Hypselotritonjiaoren*	18.3	19.5	17.8	6.6	19.3	0.6			
7	*Hypselotritonmaguae*	13.9	13.8	13.3	22.5	13.2	21.3	0		
8	*Hypselotritoncyanurus*	21.1	21.3	20.6	20.8	20.8	19.5	23.4	0	
9	*Hypselotritonyunnanensis*	19.6	20.4	20.0	20.9	20.8	20.9	22.8	11.5	2.6

Accordingly, combining the results of the morphological examination presented below and the phylogenetic analysis, the specimens from northeastern Jiangxi are regarded as a new species that is described herein.

### ﻿Taxonomic account

#### ﻿ Hypselotriton huanggangensis

Taxon classificationAnimaliaUrodelaSalamandridae

Jiang, Huang, Fan, Cheng, Raffaëlli & Chen sp. nov.

158DB4EF-DDF4-5E74-8F65-F1437DC0BE85

https://zoobank.org/18334E5C-BED9-47AE-A42D-FA916E7B2CC2

Figs 3 , 4 , 5

##### Type material.

***Holotype*.** HSA 23097, adult male from Huanggangshan Mountains (28.72°N, 117.33°E; elevation 84 m), Yanshan County, Shangrao City, Jiangxi Province, China, collected by Zhihao JIANG on 22 July 2023.

**Figure 3.**
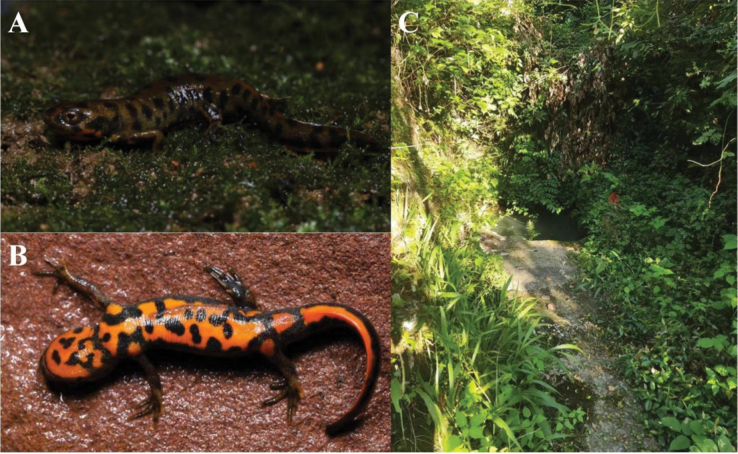
Paratypes of *Hypselotritonhuanggangensis* sp. nov. (HSA23075) **A** dorsalateral view in life **B** ventral view in life **C** small waterhole around mountain shrubs without direct sunlight at the type locality.

***Paratypes*.** Six adult males: HSA23075–23076, HSA23098–23099, HSA23102–23103, and two adult females: HSA23100–23101. Same collection date and locality as the holotype.

##### Etymology.

The specific name *huanggangensis* refers to the type locality in the Huanggangshan Mountains. For the English common name, we suggest “Huanggangshan Fire-bellied Newt” and for the Chinese name, 黄岗山蝾螈 (huáng gǎng shān róng yuán).

##### Diagnosis.

(1) Small body size, TOL 73.0–79.1 mm in adult males, TOL 79.6–89.4 mm in adult females; (2) obvious black patches with clear boundaries on the whole body; (3) ground color of the dorsal body tan; (4) skin rough; (5) ground color of venter bright orange; (6) vertebral ridge weak; (7) fingers and toes overlapping when forelimb and hindlimb adpressed towards each other along body; (8) parotoid gland inconspicuous; (9) postocular orange spot absent; (10) white warty glands around the eye; (11) two discontinuous longitudinal lines which consist of white warty glands from neck to lateral parts of tail (Fig. 6).

##### Description of the holotype.

**Figure 4.**
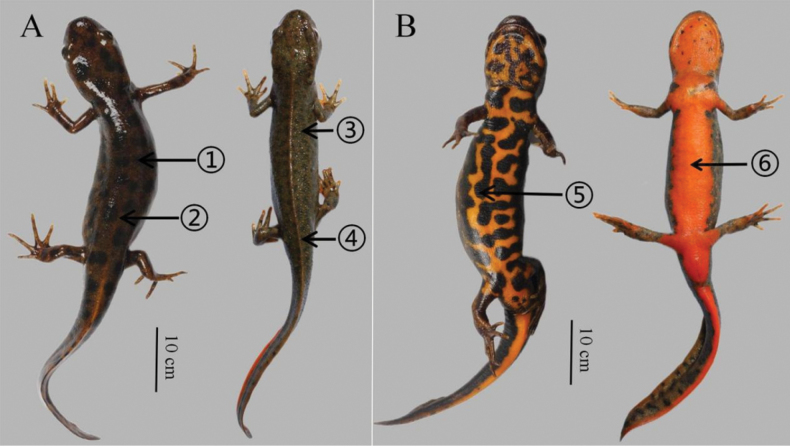
Comparison of holotype of *Hypselotritonhuanggangensis* sp. nov. (left, HSA23097) and *Hypselotritonfudingensis* (right, HSA23108) in life **A** dorsal view **B** ventral view, 1, 5 obvious black patches, 2 weak vertebral ridge, 3 small spots, 4 conspicuous vertebral ridge, 6 bright orange venter without dark blotches.

**Figure 5.**
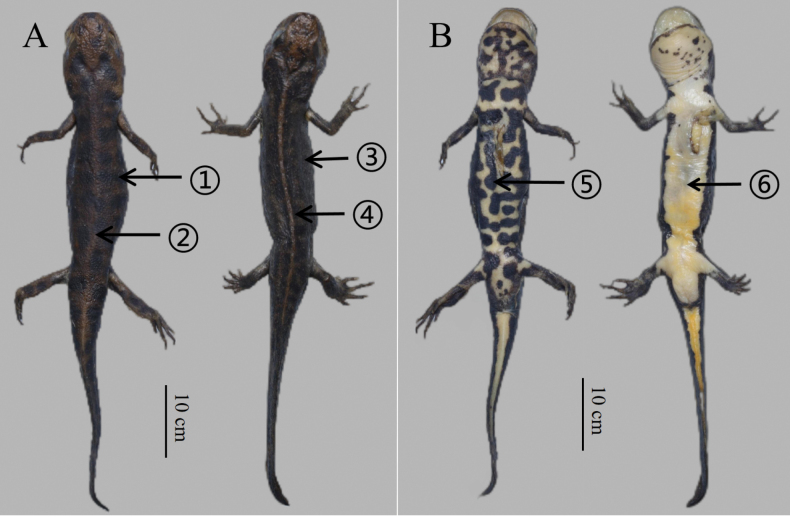
Comparison of holotype of *Hypselotritonhuanggangensis* sp. nov. (left, HSA23097) and *Hypselotritonfudingensis* (right, HSA23104) in preservative **A** dorsal views **B** ventral views, 1–6 same as in Fig. 4.

HSA 23097 (Figs 4, 5), adult male with a small, slender body (TOL 79.1 mm, SVL 47.3 mm). Head oval in dorsal view; snout truncate, projecting slightly beyond mandible; nostril small but conspicuous; tongue elongate, enlarged anteriorly, with free lateral margin; vomerine tooth patch ∧–shaped; parotoid gland inconspicuous, gill remnants absent; gular fold present; skin with fine granules, covering most parts of dorsum, venter, chin and tail; vertebral ridge weak; cloacal opening oval, slightly protruding; limbs slender, fingers and toes overlapping when forelimb and hindlimb adpressed towards each other along the body; four fingers and five toes, slender and elongated, lacking webbing; relative length of fingers I < IV < II < III; relative length of toes I < V < II < IV < III. Tail laterally compressed, tapers posteriorly; caudal fin distinct; tail tip bluntly pointed.

**Figure 6.**
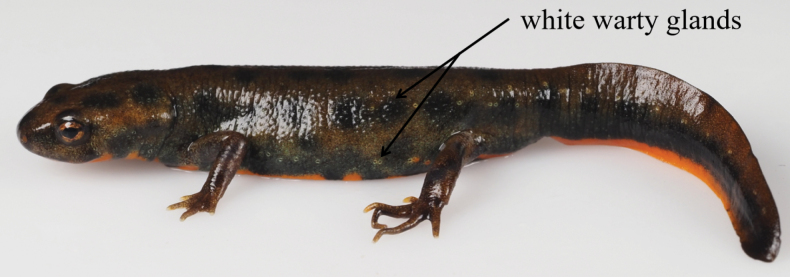
Two discontinuous longitudinal lines which consist of white warty glands from neck to lateral parts of tail (*Hypselotritonhuanggangensis* sp. nov., HSA23099) in life.

##### Coloration of the holotype.

In life, obvious black patches with clear boundaries on the whole body; ground color of the dorsal body tan; ground color of venter bright orange; white warty glands from the lateral part of head to tail; axilla, cloacal opening, and venter of tail bright orange. In preservative after six months (Fig. 5), dorsum, flanks, and limbs slightly darker. All orange coloration of venter fades to creamy white.

**Table 4.** Test for differences between *H.huanggangensis* sp. nov. and *H.fudingensis* using ANCOVA (SVL as covariate).

Variable	Males	Females
Total length (TOL)		
*F*	6.78	12.17
*P*	0.052	0.008
Tail length (TAL)		
*F*	1.09	2.27
*P*	0.419	0.185
Maximum tail depth (TAD)		
*F*	12.34	15.48
*P*	0.019	0.004
Head length (HL)		
*F*	8.76	9.66
*P*	0.035	0.013
Maximum head width (HW)		
*F*	3.77	16.71
*P*	O.120	0.004
Snout length (SL)		
*F*	1.18	1.23
*P*	0.395	0.357
Eye diameter (ED)		
*F*	12.00	1.91
*P*	0.020	0.228
Interorbital distance (IOD)		
*F*	1.20	1.48
*P*	0.390	0.301
Eye–nostril length (EN)		
*F*	0.03	0.38
*P*	0.967	0.700
Internasal distance (IND)		
*F*	5.91	0.189
*P*	0.064	0.833
Axilla–groin length (AG)		
*F*	76.89	9.38
*P*	0.010	0.014
Forelimb length (FLL)		
*F*	0.07	8.86
*P*	0.938	0.016
Hindlimb length (HLL)		
*F*	1.48	5.65
*P*	0.330	0.042

##### Variation.

Linear measurements are summarized in Table 2. Females (TOL 79.6–89.4 mm) are distinctly larger than males (TOL 73.0–79.1 mm). All paratypes resemble the holotype except that the cloaca is wider and more swollen in males than in females, the irregular bright-orange patches on ventral surface occupy more surface in females than in males, and the gular fold absent in some individuals.

##### Comparisons.

*Hypselotritonhuanggangensis* sp. nov. is phylogenetically close to *H.fudingensis*, which is distributed in northeastern Fujian. However, *H.huanggangensis* sp. nov. differs from *H.fudingensis* by its weak vertebral ridge (vs vertebral ridge conspicuous), dorsal black patches (vs small or no spots on dorsum), and ventral black patches (vs venter bright orange without dark blotches).

In addition, *H.huanggangensis* sp. nov. further differs from the remaining congeners.

*Hypselotritonhuanggangensis* sp. nov. differs from *H.orphicus* by its weak vertebral ridge (vs slightly bulged) and obvious black patches with clear boundaries on the whole body (vs small or moderate dorsal blackish dots in *H.orphicus*).

*Hypselotritonhuanggangensis* sp. nov. differs from *H.orientalis* by its parotoid gland inconspicuous (vs conspicuous) and the presence of obvious black patches with clear boundaries on the dorsum (vs absent in *H.orientalis*).

*Hypselotritonhuanggangensis* sp. nov. differs from *H.glaucus* by its obvious black patches with clear boundaries on the whole body (vs dorsum, flanks, limbs, and upper side of tail with irregular obscure greyish blue patches in *H.glaucus*).

*Hypselotritonhuanggangensis* sp. nov. differs from *H.jiaoren* by its rough skin (vs smooth), and obvious black patches with clear boundaries on the whole body (vs dorsum, flanks, limbs, and upper side of tail uniformly dark brown in *H.jiaoren*).

*Hypselotritonhuanggangensis* sp. nov. differs from *H.maguae* by having its fingers and toes overlapping when forelimbs and hindlimbs are adpressed (vs forelimbs and hindlimbs not meeting when adpressed towards each other along body), and obvious black patches with clear boundaries on the whole body (vs dorsum, flanks, limbs, and upper side of tail uniformly dark brown in *H.maguae*).

*Hypselotritonhuanggangensis* sp. nov. can be distinctly distinguished from *H.wolterstorffi*, *H.cyanurus*, *H.chenggongensis* and *H.yunnanensis* by its absent postocular orange spot (vs present).

##### Distribution and habitat.

*Hypselotritonhuanggangensis* sp. nov. is currently known only from the type locality on the western side of the Wuyishan Mountains in northeastern Jiangxi. Newts were found in a small waterhole around mountain shrubs without direct sunlight, at 84 m a.s.l. All individuals were observed in July, September, and February.

## ﻿Discussion

Despite more than a century of effort, taxonomists have yet to reach a consensus on the concept of species and methods of all species delimitation (Mayden 1997; de Queiroz 1998; Fu and Zeng 2008; Yang and Rannala 2010; Chen et al. 2013; Peng et al. 2014). Trying to solve “the notorious problem of taxonomic uncertainty (Uetz et al. 2024)”, Huang et al. (2021) proposed the principle of “species subdivision” with recommendable “subdivision” at the species level. It should help to understand species natural history more effectively and facilitate consistent actions in taxonomy and practices of conservation biology.

In the present study, although the genetic distance based on the ND2 gene fragment between *H.huanggangensis* sp. nov. and its sister species *H.fudingensis* is not very large (uncorrected *p*-distance = 2.2%), morphological differences between them are distinct (Fig. 4; Table 2). In addition, *H.huanggangensis* sp. nov. and *H.fudingensis* are isolated by a series of high mountain ranges, including the Wuyishan and Jiufengshan Mountains. These barriers often isolate different amphibian species (Chen et al. 2020).

With the addition of the new species described here, the genus *Hypselotriton* now includes 11 species. Until now, seven species of *Hypselotriton* were found in the southeastern Chinese hilly area (*H.huanggangensis*, *H.jiaoren*, *H.glaucus*, *H.maguae*, *H.orphicus*, *H.fudingensis* and *H.orientalis*), and another four species of *Hypselotriton* are distributed in southwestern China. Due to unresolved relationships, a more extensive sampling of taxa and molecular data are necessary for reliable conclusions on the evolution and taxonomy of *Hypselotriton*.

## Supplementary Material

XML Treatment for Hypselotriton huanggangensis

## ﻿Additional information

### Author ORCIDs

Zhihao Jiang https://orcid.org/0009-0004-1492-0665

Song Huang https://orcid.org/0000-0001-6786-8523

Qiangyong Fan https://orcid.org/0009-0002-5515-2143

Lin Cheng https://orcid.org/0009-0004-4674-4213

Yanan Gong https://orcid.org/0000-0002-8155-7024

Zhangbo Cui https://orcid.org/0009-0000-1501-0402

Tierui Zhang https://orcid.org/0009-0000-1869-9313

Wenjun Lan https://orcid.org/0009-0005-0690-1082

Zhian Zou https://orcid.org/0009-0001-2556-2590

Xuanzhi Huang https://orcid.org/0009-0008-2873-3640

Jean Raffaëlli https://orcid.org/0000-0003-0567-3661

Jinmin Chen https://orcid.org/0000-0001-6432-7721

### Data availability

All of the data that support the findings of this study are available in the main text.
